# The role of vitamin D status on treatment outcome among HIV- infected children receiving care in Kisumu County, Kenya

**DOI:** 10.21203/rs.3.rs-3286937/v1

**Published:** 2023-09-19

**Authors:** Maurice Songoreh Asamuka, Lilian Ogonda, Calleb George Onyango, Bernard Guyah

**Affiliations:** Maseno University; Maseno University; Maseno University; Maseno University

**Keywords:** Vitamin D deficiency, HIV Infected, ART, viral suppression, CD4 count

## Abstract

**Background:**

Kenya has a paediatric HIV burden of nearly 140,000 children, of which only 48% of those on antiretroviral therapy (ART) have achieved the desired viral suppression possibly due to vitamin D deficiency. We explored the influence of vitamin D levels on treatment outcome.

**Method:**

We performed a cross-sectional study of 196 participants aged 3 – 14 years; among them 98 HIV infected who received treatment between 2019 – 2020 in Jaramogi Oginga Odinga Teaching and Referral Hospital, Kenya. The exposure was vitamin D levels, including deficient (<20 ng/ml), insufficient (≥20 - <30 ng/ml), and sufficient (30 – 50ng/ml). The outcome was optimal immune recovery (CD4 ≥ 500 cells/mm3) and optimal viral suppression (viral load ≤ 200 copies/ml). We compared difference in means for each vitamin D category between HIV infected and uninfected using independent t-test, multiple comparisons of vitamin D levels among age categories using ANOVA and post hoc test and Pearson correlation to correlate vitamin D levels, CD4 and viral load of HIV infected children.

**Results:**

Compared with HIV uninfected, HIV infected recorded mean age ± standard deviation of10.65±2.17 years with 39(39.8%) males vs. 6.68±2.81 years with 52(53.1%) males p<0.001; and the difference in vitamin D mean levels was statistically significant [28.21 ± 6.39 infected vs.30.88 ± 6.62 uninfected] t = 2.94, df =194, p = 0.004, 95%CI (0.90 – 4.59). Among age categories, mean vitamin D varied significantly F (2,193) = 10.68, p =0.001; with higher levels observed between 1–4 years category {mean difference 4.64ng/ml, p = 0.02, [95%CI 1.49 – 7.78]} and 5–9 years category {mean difference 4.33ng/ml, p = 0.001, [95%CI 1.89 – 6.38]} as compared to 10 – 14 years respectively.

Additionally, children with optimal immune recovery recorded higher proportion of vitamin D deficiency and insufficiency (12.24% and 42.86%) as compared with sub optimally recovered 1.02% and 4.08%); while children with optimal viral suppression recorded higher proportion of vitamin D deficiency and insufficiency (8.16% and 30.61%) as compared with sub optimally suppressed (5.1% and 16.3%).

**Conclusion:**

Infections with HIV suppresses levels of vitamin D, but this has no influence on CD4 counts and viral load status in children receiving ART.

## Introduction

About 40% of the 1 million HIV infected children < 15 years of age, receiving antiretroviral treatment (ART) care in sub Saharan Africa have not achieved the desired viral suppression *(defined as viral load < 200 copies /ml)* despite improved implementation of more efficacious 1st line ART regimens [1], possibly due to vitamin D deficiency. Plasma vitamin D *[defined as 25 hydroxyvitamin D (25(OH)D]* is a steroid hormone, fat-soluble micronutrient obtained primarily from ultraviolent B rays and plays crucial roles of balancing homeostasis, regulating mineral metabolism, bone maintenance, cell growth, and immune response useful in containing infectious diseases [2–5]. Deficiency of vitamin D therefore, promotes inflammatory responses by upregulating inflammatory markers such as IL-6 and TNF-α overproduction in HIV-infected patients [2, 6], leading to renal 1*α*-hydroxylase impairment with reduction of parathyroid hormone (PTH) stimulatory effect on the production of hormonally active 1,25 dihydroxyvitamin D [1,25(OH)2D] [7]. Additionally, vitamin D deficiency accelerates faster progression of HIV disease and perfectly correlates with decreased patient survival [3, 8].Thus, there is a need for periodic evaluation of levels with subsequent supplementation where necessary during infections and treatment.

Conversely, the body immune system such as the brain, heart, stomach, lymphatic system, skin, and prostrate tissue are composed of CD4^+^ T-cells and B-cells that express vitamin D receptors (VDR) useful in improving immune functions and reduction of inflammations [9, 10]. Interactions between VDR and Vitamin D mediate anti-inflammatory effects on both innate and adaptive immune systems that regulate immunity in the context of bacterial or viral inflammation [10]. Consequently, severe vitamin D deficiency is linked to low CD4 + cell counts and increased markers of inflammation in HIV-infected patients [5, 11] suggesting a possible relationship among vitamin D, infectious diseases and immune functions in pediatric population that highlights the role and need for supplementation in cases of deficiencies [10, 12].

Moreover, studies have also shown that HIV infected participants on ART, and who received vitamin D supplementation to sufficient levels recorded much improved viral suppression compared to their counterparts who experienced low levels of vitamin D [13]; thus, suggesting a possible influence of vitamin D on treatment outcome.

Kenya has nearly 140,000 children living with HIV (CLHIV) < 15 years of age and 8000 new pediatric infections annually, but only 48% of those on ART have achieved the desired viral suppression [14]. In Kisumu County, adolescents and young adults living with HIV (AYALH) has continued to register suboptimal viral suppression of 84.4% overtime despite increasing availability of improved services [15], and vitamin D deficiency has been suggested as a potential risk factor driving this poor outcome [16]. This study explored the correlation between vitamin D levels, immune status and viral suppression as prognosis indicators in children receiving ART in Kisumu County

## Method

Using *Sharma 2019* formula, blood samples of 196 [98 HIV infected] consenting participants aged 3–14 years from Kisumu County was determined for a cross-sectional study by systematic sampling. Laboratory tests were conducted at Jaramogi Oginga Odinga teaching and referral hospital (JOOTRH), Kenya from November 2019 to March 2020 based on large volume of participants receiving comprehensive care, and quality standards available. Permission to conduct study was obtained from JOOTRH ethical review committee, and participants were enrolled upon receipt of guardian informed written consent.

Children on vitamin D supplements were excluded from the study. Demographic details were extracted from clinic records by nurse counselor who equally collected 2ml of venous blood sample into each plain and EDTA vacutainer tubes *(BD Franklin Lakes, USA)* for evaluation of viral load, vitamin D and CD4 + cells levels, respectively.

Sera were tested for vitamin D using Biomerieux^®^ Mini Vidas automated immunoassay analyzer, and HIV-1 RNA using Roche diagnostics^®^ COBAS real time PCR test, while, CD4^+^ cell count was performed using Becton Dickinson^®^ fluorescence-activated cell sorter (FACS) system. All tests were done according to manufacturers’ instructions. Statistical analysis was performed using SPSS version 20.

We compared difference in means for each vitamin D category between HIV infected and uninfected using independent t-test, multiple comparisons of vitamin D levels among age categories using ANOVA and post hoc test and Pearson correlation to correlate vitamin D levels, CD4 and viral load of HIV infected children.

## Results

Overall, we recruited 196 participants of which 98 were HIV infected age range 5–14 years with another 98 HIV uninfected (control group) age 3–14 years. HIV infected participants recorded a mean age ± standard deviation of 10.7 ± 2.17 years with a median (IQR) duration of ART of 6.2 years (3.1–13.2), 39.8% being male, and a mean vitamin D level of 28.21 ± 6.39; as compared to uninfected group having a mean age ± standard deviation of 6.7 ± 2.81, 53.1% being males and a mean vitamin D level of 30.88 ± 6.62. Overall, the difference in mean vitamin D between HIV infected and uninfected participants was statistically significant [t = 2.94, df 194, p = 0.004 (Table 1). However, using vitamin D deficiency, insufficiency and sufficiency cut-off values of < 20 ng/ml; ≥20 - <30 ng/ml; and 30–50ng/ml respectively, HIV infected participants recorded mean deficiency, insufficiency and sufficiency levels of 18.1ng/ml, 25.9ng/ml, and 34.3ng/ml as compared to 18.4ng/ml, 25.5ng/ml and 35.7ng/ml respectively, for the uninfected group with no statistical differences.

**Figure 1:**
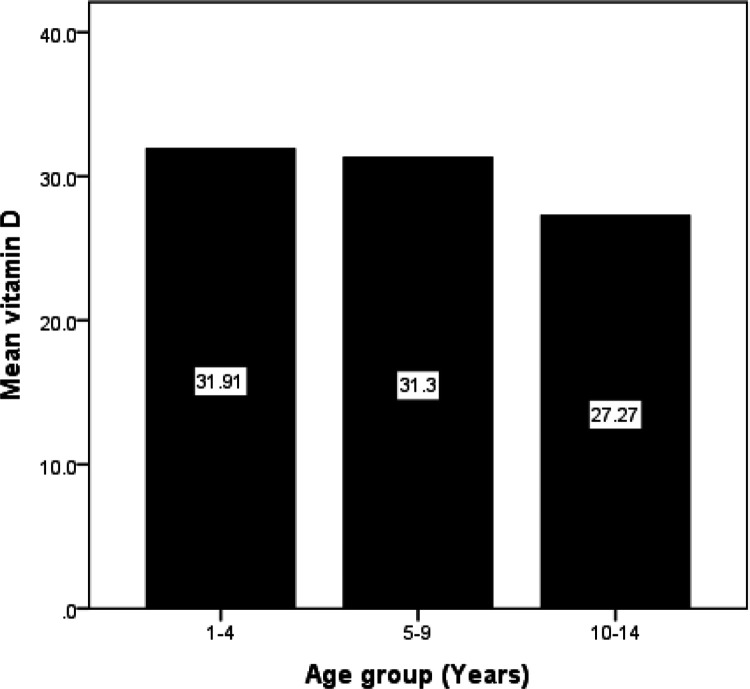
Show mean proportions of vitamin D levels in different age categories.

We performed multiple comparisons of vitamin D levels among age categories [(1–4), (5–9), (10–14) years using ANOVA and post hoc test at 95% confidence interval and found the difference was statistically significant p < 0.05; F (2,193) = 10.68, p = 0.001 (Table 2). Further analysis by Turkey-HSD post hoc test revealed that age group 1–4 yrs. had significantly higher vitamin D levels {mean difference 4.64, p = 0.02, [95%CI 1.49–7.78]} compared to group 10–14 yrs. Additionally, group 5–9yrs showed significantly higher vitamin D levels {mean difference 4.33, p = 0.001, [95%CI 1.89–6.38]} compared to 10–14 yrs. But there was no significant difference between group 1–4 and 5–9 yrs. (Fig. 1). Pearson correlation coefficient establish no correlation between vitamin D levels, CD4, and viral load ( Table 3).

However, we examined the clinical outcomes of the two categories of HIV infected participants (Immunocompromised defined as CD4 count < 500 cells/mm3 visa-a-vie Immunocompetent defined as CD4 count ≥ 500 cells/mm3) by comparing their vitamin D status. From our results, higher proportion of vitamin D deficiency and insufficiency occurred among Immunocompetent participants as compared with immunocompromised group [12.24%, 42.86% vs.1.02%, 4.08%] respectively. Equally, we examined the clinical outcomes of participants with optimal viral suppression (defined as viral load < 200 copies / ml) visa-a-vie participants with sub optimal suppression (defined as viral load ≥ 200 copies / ml) by comparing their vitamin D levels. Our finding showed that higher proportion of vitamin D deficiency (8.16%) and insufficiency (30.61%) occurred among the optimally suppressed group as compared to the sub optimally suppressed group (8.16%, 30.61% vs. 5.1%, 16.3%) respectively (Table 4).

Table 3 shows numbers (n) and proportions (%); Optimal viral load suppression (< 200); High level viremia (≥ 200); Low CD4 counts (< 500) and Optimal CD4 count (≥ 500). There is no correlation between vitamin D concentration and viral suppression or immunocompetence

## Discussion

Although 25(OH) D levels are commonly used to define vitamin D status, its suitability and applicability as a predictor for good prognosis in children on HIV care remains debatable. In our study, we examined the relationship between age categories and levels of vitamin D. Our results revealed that levels of vitamin D in children increases with increasing age during1–4 years to reach peak levels of 31.91ng/ml and begin to decline (Fig. 1). Supposedly, this value represents the physiologically ideal level of vitamin D much required to optimize various vitamin D high level viremia-associated health outcomes and physiological parameters such as bone mineral density [17]; and is perfectly comparable with 36.5ng/ml peak levels at 18 month postnatal earlier reported in a Malawi study [18], Furthermore, our finding is supported by Mogire *et al* [19] and Alvarez-Rodriguez *et al* [20] who equally observed a decreasing trend with vitamin D levels as age increases; and associated the same with a change in expression and defective function of some toll-like receptors (TLRs) involved in viral response [20]. To the contrary, a similar study among HIV uninfected infants in our neighborhood Tanzania observed that low levels of vitamin D was common in infants during their early infancy, especially those under exclusive breastfeeding.[21], the cause of which remain unclear.

Furthermore, we compared the mean levels of vitamin D between HIV infected and uninfected children receiving antiretroviral therapy (ART) with those on routine outpatient visits. Our data analysis revealed that HIV infected children profoundly experienced low levels of vitamin D as compared to their uninfected counterparts. Interestingly, this is explained by the fact that persons living with HIV (PLWH), and receiving ART particularly protease inhibitor (PIs) or non-nucleoside reverse transcriptase inhibitors (NNRTIs) traditionally experience suppressed levels of vitamin D in blood [22], but which becomes more exacerbated in individuals receiving efavirenz (EFV) [5, 23]; the mechanism of which is under investigation. Moreover, much lower levels of serum vitamin D have equally been observed among late ART-initiated children (18 months to 12 years) as compared to those on early-ART initiation in the first year of life [22], but this is explained by the influence of increasing age on vitamin D levels reported in our study; and is consistent with children physiological parameters such as bone mineral density [17].Thus, our finding supported an earlier systematic review and meta-analysis report showing increased risk of vitamin D deficiency in PLWH compared to uninfected subjects; more so to participants receiving ART, and in older age category [24]. Slightly lower levels of vitamin D among HIV infected infants and adults on ART have equally been reported in a similar study in Botswana [4], although this was partly attributed to demographic and dietary habits of consuming more of traditional and indigenous foods enriched with better dietary diversity [4]

Additionally, we compared the proportion of Immunocompromised participants with Immunocompetent group based on their vitamin D levels, and established that majority participants who experienced vitamin D deficiency or insufficiency were actually Immunocompetent rather than Immunocompromised; and consistent with earlier findings by Kakalia et al, [25]. To the contrary, our findings dispelled earlier report suggesting a possible correlation between vitamin D levels and CD4 + T-cell counts [3, 5, 26]. Instead, vitamin D concentrations above 30 ng/ml has been found to be more associated with mortality of HIV infected children compared to levels lower than 10 ng/ml [21]. Meanwhile, a good number of local and international studies have reported varying relationship; both positive and negative, with others failing to demonstrate any correlation [27] A recent study in the neighborhood Uganda associated low CD4 count with vitamin D deficiency [5]; thus, echoing other findings from related studies in France and Colombia which equally suggested that severe vitamin D deficiency could be independently associated with low CD4 count [3, 4, 28]. Although high CD4 cells counts have been associated with higher vitamin D levels [29], while low vitamin D levels is linked to immune dysfunction that also influence the expression of inflammatory markers [24], we could not establish this outcome owing to the scope of our study. Moreover, it is good to note that children in our study had been on ART for quite some times, majority of whom were experiencing good health status by the time of sampling; hence, the immunologic, HIV stage and other HIV related factors may have normalized and compensated for the effects of vitamin D status. We however still believe that this could have been a bit different in children who may have had advanced disease at ART initiation.

Moreover, we compared the proportions of participants who achieved optimal viral suppression visa-vi those with sub optimal suppression and their levels of vitamin D; and found that those who were optimally suppressed were in fact the majority with either vitamin D deficiency or insufficiency contrary to earlier suggestion by Stalling *et al* that vitamin D supplementation intervention to sufficient levels helps to improve viral suppression of patients on ART[13] Our findings equally dispute earlier suggestion by Overton et al that high dose supplementation of vitamin D with ART substantially enhance viral suppression [30].

This study included a number of limitations. First, the small sample size and single site experience limits generalizability and requires further study in multisite samples. Additionally, our study was performed entirely in Kisumu western Kenya, an equatorial region abundant with sunshine which may have compensated for deficiency attributed to diet or HIV infection; thus, generalizability to other regions with less sunshine may be limited. Finally, our population consisted of children with relatively preserved immune function, having been on ART for sometimes and therefore our failure to accurately demonstrate the relationship between vitamin D status, CD4 count and viral load; hence, cannot be generalized to children with more advanced HIV disease and low baseline CD4 counts.

## Conclusion

In early childhood, vitamin D increases with increasing age to optimal levels by age 5–9 years during which it stabilizes and begin to decrease with advancing age. Our data indicate that infections with HIV disproportionately influence levels of vitamin D in children, although this has no effect on CD4 counts and viral load. We recorded a low prevalence of vitamin D deficiency and insufficiency in a small cohort, and suggest that a further study with ART naïve participants with larger sample size would be ideal to better understand possible confounding variables that may affect vitamin D status in HIV infected children

## Figures and Tables

**Table 1:** Demographic characteristics of the study participants Data shows numbers (n) and proportions for each group.

		HIV infected (n = 98)	HIV uninfected (n = 98)	p value
Age(years)	Mean	10.65± 2.17	6.68 ± 2.81	< 0.001^a^
Gender	Male	39(39.8%)	52(53.1%)	
	Female	59(60.2%)	46(46.9%)	
Vitamin D (ng/ml)	Mean	**28.21 ±6.39**	**30.88 ± 6.62**	**0.004^a^**
	Deficient (≤ 20ng/ml)	13(13.3%)	5(5.1%)	
	Mean	18.1	18.4	0.733^a^
	Insufficient (21–30)	46(46.9%)	37(37.8%)	
	Mean	25.9	25.5	0.575^a^
	Sufficient (≥ 30ng/ml)	39(39.3%)	56(57.1%)	
	Mean	34.3	35.7	0.075^a^

a = independent t test

**Table 2:** Multiple Comparisons of vitamin D levels among age categories based on ANOVA and post hoc test

Dependent Variable: vitamin D						
Turkey HSD						
(I) Age group (Years)	(J) Age group (Years)	Mean Difference (I-J)	Std. Error	Sig.	95% Confidence Interval	
					Lower Bound	Upper Bound
(1–4)	(5–9)	.6026	1.3556	.897	−2.599	3.804
	(10–14)	4.6360*	1.3310	.002	1.492	7.780
(5–9)	(1–4)	−.6026	1.3556	.897	−3.804	2.599
	(10–14)	4.0334*	.9945	.000	1.685	6.382
(10–14)	(1–4)	−4.6360*	1.3310	.002	−7.780	−1.492
	(5–9)	−4.0334*	.9945	.000	−6.382	−1.685

*. The mean difference is significant at 0.05 level.

**Table 3:** correlations between Vitamin D, CD4 and viral load (Pearson correlation)

		CD4	VIRAL LOAD
**VITAMIN D**	Correlation coefficient	0.166	−0.115
N = 98	significance 0.01 (2 tailed)	0.101	0.26

Significance is at 0.01 level

**Table 4:** Clinical characteristics of the study participants

Vitamin D n(%) = 98		Deficient (< 20 ng/ml)	Insufficient (>20 – <30)	Sufficient (30–50)
Optimal suppression (VL- copies/ml)	<200	8(8.16%)	30(30.61%)	29(29.59%)
High level viremia (VL- copies/ml)	≥200	5(5.1%)	16(16.3%)	10(10.20%)
Immunocompromised CD4(cells/mm3)	<500	1(1.02%)	4(4.08%)	1 (1.02%)
Immunocompetent CD4(cells/mm3)	≥ 500	12(12.24%)	42(42.86%)	38(38.78%)

## Data Availability

The dataset supporting the findings of this study is available as a supplementary material.

## Abbreviations

CI: confidence interval
SD: standard deviation
CDC: center for disease control and prevention
ART: antiretroviral therapy
25(OH)D: *25 hydroxyvitamin D*
1,25(OH)2D: 1,25 dihydroxyvitamin D
VDR: vitamin D receptors
CLHIV: children living with HIV
AYALH: adults and young adults living with HIV
VL: viral load
